# Dietary Intake of Protein and Essential Amino Acids for Sustainable Muscle Development in Elite Male Athletes

**DOI:** 10.3390/nu15184003

**Published:** 2023-09-15

**Authors:** Marius Baranauskas, Ingrida Kupčiūnaitė, Rimantas Stukas

**Affiliations:** 1 Faculty of Biomedical Sciences, Panevėžys University of Applied Sciences, 35200 Panevėžys, Lithuania; ingrida.kupciunaite@panko.lt; 2 Institute of Health Sciences, Faculty of Medicine, Department of Public Health, Vilnius University, 01513 Vilnius, Lithuania; rimantas.stukas@mf.vu.lt

**Keywords:** nutrition, diet, elite athletes, anaerobic sports, aerobic sports, body composition, muscle development, protein, essential amino acids

## Abstract

Athletes need to develop a relatively high muscle mass and low body adipose tissue for the sake of better athletic performance. A full range of nine essential amino acids and eleven non-essential amino acids have to attend in appropriate amounts for protein biosynthesis. The aim of the observational comparative cross-sectional study was to assess the association between the diet quality profile and training-induced muscle mass estimated by bioelectrical impedance among elite male athletes. The research sample comprised 18.1 ± 3.1 year-old Lithuanian professional male athletes (n = 234). The study participants were enrolled to complete 24-h dietary recalls of three non-consecutive days. The body composition was assessed using the bioelectrical impedance analysis (BIA) method. The present study showed a significant insufficiency of the mean carbohydrate intake of 5.7 g/kg/day in a group of aerobic male athletes. The lower muscle mass of aerobic male athletes was related to the lower-carbohydrate diet (adjusted odd ratio (OR_adj_) 0.3; 95% confidence interval (CI): 0.1–0.7). The mean protein intake of 1.8 g/kg/day was optimal for anabolism in the samples of both anaerobic and aerobic male athletes. The protein intake in appropriate doses was potentially associated with an increase in muscle mass only in anaerobic male athletes (OR_adj_ 2.2; 95% CI: 1.3–3.7). The positive relationship was revealed between the possible muscle mass gain and the increased intakes of amino acids such as isoleucine and histidine among anaerobic athletes (OR_adj_ 2.9; 95% CI: 1.1–4.7 and OR_adj_ 2.9; 95% CI: 1.0–4.3, respectively). An inverse feasible association was indicated between a higher intake of valine and lower muscle mass quantities among anaerobic male athletes (OR_adj_ 0.1; 95% CI: 0.1–0.5). The recommendations for sports nutritionists should emphasize the necessity of advising professional athletes on dietary strategies on how to manipulate dietary amino acid composition with respect to achieving long-term body composition goals.

## 1. Introduction

Nutrition is characterized as a certain behavior by which nutrients are consumed in sufficient amounts for ensuring a good standard of living and maintaining a healthy lifestyle. The daily nutritional requirements for athletes are particularly strict. More specifically, athletes must consume nutrients in increased and balanced quantities. In addition, the nutritional profile of athletes is important for optimizing athletic performance and depends on factors, namely sex, age, branch of sports, and athletic goals, as they appear to be related to body composition [1]. Athletes need to develop a relatively high muscle mass and low body adipose tissue for the sake of better athletic performance. The striated muscle tissue is the most metabolically active bodily tissue. Muscle proteins are unstable, as they have a permanent ability to turn over, i.e., degrade and synthesize. The changes in both the synthesis and degradation of muscle proteins play an important role in the recovery and remodeling of muscle proteins following physical loading. The changes in muscle mass depend on the changes in the protein content in myofibril, accordingly. Thus, such metabolic regulation has a key role in the adaptations of skeletal muscle (in terms of size) to exercise training. Approximately 5% of essential amino acids (EAAs) from protein catabolism are oxidized in muscles at partial levels and inaccessible for a new protein translation. In all, 25% of EAAs released into the blood are used by other body tissues. The remaining amount (70%) of essential amino acids is recycled into protein synthesis [2]. Thus, protein degradation rates always surpass the biological process of the synthesis of new protein cells by 30% on average, depending on an insufficient EAA intake.

The higher rate of protein synthesis can only occur with protein intake in appropriate doses [3]. It has been found that, due to aminoacidemia, the positive muscle protein net balance can be ensured between 1.5 and 3 h after consumption of a high-protein meal within 30 to 45 min during the postprandial state [4]. In addition, the amino acid-mediated anabolic stimulation of muscle protein synthesis relies on the amount of dietary protein and amino acid intake, i.e., the biological value of proteins. There are a total of 20 amino acids that make up muscle proteins. Nine out of these 20 amino acids belong to the group of EAAs. EAAs cannot be produced by the human body in physiologically significant quantities, and, therefore, are necessarily obtained through an adequate diet. The remaining 11 amino acids are categorized as non-EEAs, as they might be produced by the physical body [5,6]. Thus, the full range of nine EAAs and the 11 non-essential amino acids should be available in appropriate amounts for protein biosynthesis [7]. Hence, the creation of new proteins may be restricted by a limited presence of any EAA, whereas the lack of non-EAAs could be offset by the boosted de novo synthesis [8].

On the other hand, an essential variable that can be related to the general efficacy of protein/amino acids in the course of exercise training is the absolute protein intake per day. The factors associated with protein timing and the quality of protein used are equally relevant to assessing the effects of dietary protein intake during long-lasting exercise participation [9]. Therefore, a large number of meta-analyses and experimental and observational studies without intervention have been conducted to summarize the effects of the consumption of protein supplements on the occurrence of changes in body composition, muscular power and strength or the levels of bodily adaptation to exercise [10,11,12,13,14,15,16,17,18,19,20].

However, the research has focused on protein supplementation in the physically active adult population, whereas the diet quality, nutrient intake, and amino acid composition in the subjects’ diets were uncontrolled.

Scientific attention should also focus on the nutritional profile when athletes lack energy/carbohydrates and protein in their diet, as both the total daily protein intake and the quality of protein intake can be useful for the increase in and growth of muscle cells during exercise training. This scientific theory is supported by meta-analysis, which explains that the total protein consumption, with the modern Western diet, is a more important factor than the daily distribution of protein intake in triggering muscle hypertrophy during physical training [15]. Therefore, further determination of the nutritional status and body composition in athletes remains necessary, as no specific agreement has been reached on the results of protein intake to boost muscle hypertrophy. Additionally, so far there have been no studies aiming to summarize the association between the muscle mass (in terms of size) of elite athletes and the nutrient intake in most countries of the European Union and the Baltic States. The aim of this study was to assess the association between the diet quality profile and the training-induced muscle mass estimated by bioelectrical impedance among elite male athletes.

## 2. Materials and Methods

### 2.1. Data Collection and Study Participants

**Figure 1:**
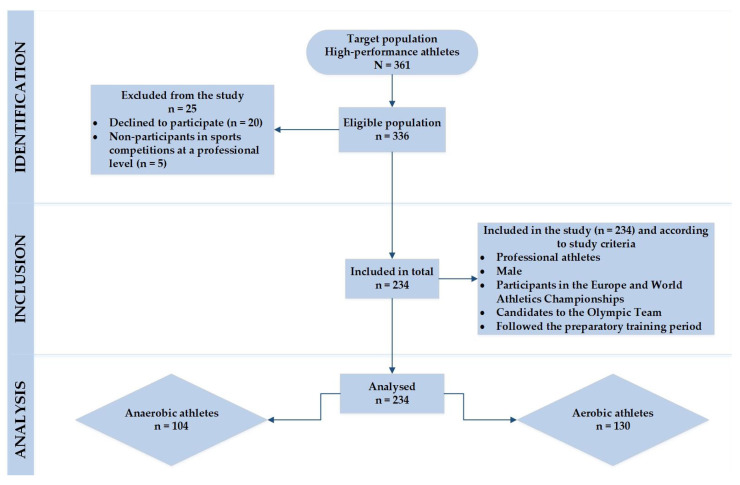
Enrollment flowchart for elite male athletes.

A total of 336 Lithuanian elite athletes of the eligible population were selected from the list approved by the Lithuanian National Olympic Committee (LNOC). Stratified random sampling was used to enroll elite male athletes for the observational study. The following requirements were the main criteria for inclusion of athletes in the study: (1) professional male athletes participating in the preparatory training period; (2) participants in the Europe and World Athletics Championships; (3) candidates for the Olympic Team. A more detailed algorithm for the recruitment process is presented in Figure 1.

Finally, during the period from 2018 to 2019, 234 elite male athletes aged 18.1 ± 3.1 years involved in exercises for Olympic sports, namely boxing, judo, Greco-Roman wrestling, taekwondo, weightlifting, basketball, gymnastics, high jump, rowing, road cycling, swimming, skiing, biathlon, long-distance running, and modern pentathlon, were included in the comparative cross-sectional study (Table 1).

The Lithuanian elite male athletes engaged in different sports were classified into two groups, namely anaerobic (75.3%, n = 104) and aerobic (65.7%, n = 130) athletes, depending on two primary energy-producing pathways in the body [21]. All tests related to the study were performed during the preparatory training period prior to the competitions in a cohort of male elite athletes. The average physical load duration in study participants matched 176.9 ± 62.6 min per day. The physical activity levels of elite male athletes fully conformed to the intensity zones approved for training plans by the Lithuanian Sports Centre (LSC) and LNOC (Table 2).

### 2.2. Anthropometric Measures

The analysis of the body composition of elite athletes was conducted at the Lithuanian Sports Medicine Centre (LSMC). A stadiometer was used for measuring the height to the nearest ±1 cm of athletes. Due to the bioelectrical impedance analysis (BIA) conducted as a non-invasive testing method referring to the third level of validity approach, the outcomes of which were highly correlated to those of dual-energy X-ray absorptiometry (DXA) [22,23], it was possible to analyze and estimate the body composition using the electrical resistance of various tissues of the body [24,25]. In 234 male athletes, the body composition evaluation using the BIA method via an X-scan (Kyungsan City, Republic of Korea) device was conducted. Impedance measurements were performed by using 5 different electrical signals of 5, 50, 250, 550, and 1000 kHz. The BIA provided information on the body composition of athletes: body weight (BW) (kg), fatty tissue mass (FM) (kg and percentage of BW), lean body mass (LBM) (kg and percentage of BW), muscle mass (MM) (kg and percentage of BW). The muscle and fat mass index (MFMI) of each athlete was calculated by dividing MM (kg) by BF (kg). For BIA outcomes and the assessment of individual body mass components (FM, MM, and MFMI), appropriate scales were used. These norms identified for elite athletes were previously invented, published, and currently used by the authors for the aims of this study [26].

### 2.3. Nutritional Assessment

The resting metabolic rate (RMR) was estimated using the Harris–Benedict equation [27]. Twenty-four-hour physical activity recalls were gathered on the same day the athletes reported for their daily calorie consumption. The additional energy expenditure during exercise (training energy expenditure (TEE)) was estimated in the light of the recommendations of the American Dietetic Association, Dietitians of Canada, and the American College of Sports Medicine [28] as well as the report of empirical research by Ainsworth and colleagues [29].

Considering the fact that there is currently no “gold standard” for assessing dietary intake, dietary recalls, as the most common approach used in sports nutrition research [1,30,31,32,33,34,35], were also applied in our study. The 24-h dietary recall method was used in the dietary assessment of athletes [28,30,36,37]. The participants were instructed to maintain their habitual diet throughout the study and finally were enrolled to complete the forms for their 24-h dietary recalls of three non-consecutive days with the assistance of a sports dietitian [38]. The forms with dietary recalls were collected from all participants by a sports dietitian using a direct personal interview method during the physical examination of athletes at the LSMC. All food and drinks consumed by the study participants were recorded by the trained interviewer in line with the basis of the amounts of food provided in the Atlas of Foodstuffs and Dishes [39]. A list of the athletes’ average daily food intakes was compiled during the next data processing phase. The nutritional analysis software NutriSurvey (the English translation of a professional German nutrition software program (EBISpro)) was applied for tracking the ingredients used in food recipes in order to calculate the nutrition values of individual food items (http://www.nutrisurvey.de/ (accessed on 5 January 2018)). Additionally, the NutriSurvey function "Food/Include more foods from other databases" was used as well as the nutrition values of food items manually integrated from the Lithuanian food database [40].

Each sport discipline is based on the origin of training and is related to one of two primary energy-producing pathways in the body along with nutrient requirements. Therefore, sports nutritionists working with athletes involved in different sports (anaerobic and aerobic) have been provided with nutrition guidelines established by the international sporting committees, namely, the International Society of Sports Nutrition (ISSN), the International Olympic Committee (IOC), and the American College of Sports Medicine (ACSM). Consequently, the intakes of carbohydrates and protein in the athletes we studied were assessed in accordance with the recommended values provided in the these scientific recommendations [41,42,43,44,45,46]. More specifically, the carbohydrate content recommended for athletes is consistent with 7–10 g/kg/day. The protein intake for athletes should be within the limits of 1.4 to 2.0 g/kg/day [47,48]. Taking into account that some uncertainty remains over the adult EAA requirements, the World Health Organization (WHO) has proposed a recalculation of the individual amino acid requirements divided by the total protein requirement. In this context, the EAA requirements for athletes were assessed in accordance with the recommendations made by the WHO, following an empirical recalculation in accordance with the standards applicable to athletes [49,50]. Additionally, the widely used technique for nutritional assessment was applied for estimating the nitrogen balance (NB) in male athletes. The NB of all of the athletes was calculated using the formula: NB (g/N/day) = DNI − UNA − NUN − UNPL, where (1) DNI—dietary nitrogen intake estimated as dietary protein intake (DPI)/6.25 [51]; (2) UNA—urea nitrogen appearance; (3) NUN—non-urinary nitrogen excretion (e.g., ammonia, uric acid, creatinine, amino acids) [52,53] estimated as 31 mg/kg [54]; (4) UNPL—urinary nitrogen protein losses (estimated as 2 g for gastrointestinal and integumentary (dermal) losses) [55]. The data were rearranged to empower the estimation of UNA from protein intake (using Bergstrom’s formula, UNA = (DPI − 19)/7.62) [56].

In the meantime, fat intake may fluctuate between 20% and 35%, contingent on the daily caloric consumption by athletes.

### 2.4. Statistical Analysis

The cross-sectional study was conducted in accordance with the Strengthening the Reporting of Observational Studies in Epidemiology (STROBE) checklist [57].

Statistical analysis of the empirical data of the study was executed using both Statistical Package for the Social Sciences (SPSS) V.25 for Windows (Armonk, NY, USA) and Stata version 12.1 (StataCorp, College Station, TX, USA). The Shapiro–Wilk *W* test was used to check the normality of the data. If the data normality was confirmed, the paired two-sample *t*-tests were used to assess the significance of the differences between the central tendency measures (mean ± standard deviation (SD)) of the two categories. Cohen’s d (*d*) as an effect size was also evaluated to accompany the reporting of *t*-test results as well as to indicate the standardized difference between the two means. The effect sizes were interpreted as follows: 0.2 ≤ *d* < 0.5 (small effect), 0.5 ≤ *d* < 0.8 (moderate effect), and 0.8 ≤ *d* (large effect). If *d* was larger than 1, the difference between the two means was larger than one SD, and anything larger than 2 signified that the difference was larger than two SDs [58]. The Pearson correlation coefficient (*r*), which is the most common way of measuring a linear correlation, was used to set up the relationship between the variables such as dietary protein intake, nitrogen balance, and muscle mass in a sample of male athletes. The correlation coefficients were interpreted as follows: 0 ≤ *r* < 0.3 (negligible correlation), 0.3 ≤ *r* < 0.5 (moderate correlation), and 0.5 ≤ *r* < 1 (high correlation). All of the reported *p*-values were developed on two-sided tests and compared to the significance level of 5%.

In addition, multiple logistic regression analyses with a binary dependent variable among two subgroups covering the anaerobic and aerobic sports were performed. The dependent variables, namely muscle mass (MM) (in kg) of anaerobic athletes (61 kg ≤ MM > 61 kg) and MM (in kg) of aerobic athletes (57 kg ≤ MM > 57 kg), were adjusted to the dichotomous form (1—MM was below median value in the subgroup (reference category), 2—MM was equal to or larger than the median value in the subgroup). The units of measurement of independent variables, namely the intake of energy (kcal/kg/day), macronutrients (g/kg/day), and EAAs (mg/g of protein/day), were adjusted to the dichotomous form relying on cut-off values that were determined using the median values of the subgroups of anaerobic and aerobic male athletes. The logistic regression models were adjusted for the age and training experience of professional male athletes. Goodness-of-fit of logistic regression models was evaluated using the Nagelkerke R^2^ statistic.

## 3. Results

### 3.1. Body Composition of Elite Male Athletes

Body composition is a physical measurement that provides more specific information about physical fitness than BW alone. Body composition can be defined as the proportion of fat and muscle mass (MM) in the body. The body composition of Lithuanian elite male athletes was assessed as indicated in Table 3. LBM and MM in male athletes fluctuated within the normal range. Despite the sporting discipline, it was also found that the measures of central tendency for LBM were significantly higher than the mean norm values in both anaerobic and aerobic athletes. The ∆ LBM_in%_ (actual LBM_in%_—avg. recommended LBM_in%_) values for anaerobic and aerobic athletes were 3.3% (95% PI: 2.2; 4.3, *d* = 0.6) and 3.3% (95% PI: 2.6; 4.1, *d* = 0.8), respectively. The mean BF values for male athletes involved in both anaerobic and aerobic sports (16.7 ± 5.2% and 16.7 ± 4.2%) exceeded the levels of optimum values (10–14%) by 4.7% and indicated only acceptable body fat levels (*d* = 0.2 and *d* = 0.2, respectively). However, irrespective of relatively higher study participant BF levels, MFMI was extensive in male athletes. The *t*-tests between MFMIs in anaerobic and aerobic male athletes (5.3 ± 2.4 and 5.2 ± 2.6, respectively) and the avg. extensive MFMI limit (4.7–6.0) were found to be without statistical significance: ∆ MFMIs (actual MFMI—avg. extensive MFMI) of anaerobic and aerobic athletes were −0.1 (95% PI: −0.5; 0.4, *d* = 0.02) and −0.2 (95% PI: −0.6; 0.3, *d* = 0.05), respectively.

### 3.2. Nutritional Profile of Elite Male Athletes

Table 4 shows the reported usual mean intake of energy, carbohydrates, fats, proteins, and amino acids of the elite male athletes. Regardless of sport, the mean fat energy of 40 ± 7.6% found in the diets of male athletes exceeded 35% of calories. The carbohydrate-deficient diet (5.7 ± 0.6 g/kg/day) was usual only among aerobic athletes: ∆ carbohydrates actual intake—avg. recommended) of aerobic athletes was −2.9 (95% PI: −3.2; −2.5, *d* = 4.7). Meanwhile, the mean protein intake of 1.8 ± 0.6 g/kg/day was within the recommended limits of 1.4–2.0 g/kg/day in both anaerobic and aerobic male athletes. Consequently, in terms of effect size (*d*), it can be affirmed that the reported intakes of EAAs such as leucine, isoleucine, valine, phenylalanine plus tyrosine, methionine, lysine, tryptophan, threonine, and histidine were within plus 2 standard deviations around mean requirements and revealed over-consumption of all EAAs in the population of elite male athletes [45]; thus, these rates could be assigned to large effect sizes (2.6 ≤ *d* ≤ 6.3).

Furthermore, if an amino acid such as leucine acts as a stimulating precursor for muscle protein synthesis and has higher upper limits for recommended daily allowances (up to 12 g/day) [47,48,50], the means of leucine intake for anaerobic and aerobic athletes were calculated as follows: 10.2 ± 4.5 g/day and 10 ± 3.3 g/day, respectively.

Also, our study confirmed that the mean 24-h nitrogen balance of 2.0 ± 1.3 g in athletes was positive and related to a sufficient protein intake or consumption of more protein than the body needs, and was also optimal for anabolism.

### 3.3. Association between Protein Intake and Muscle Mass

**Figure 2:**
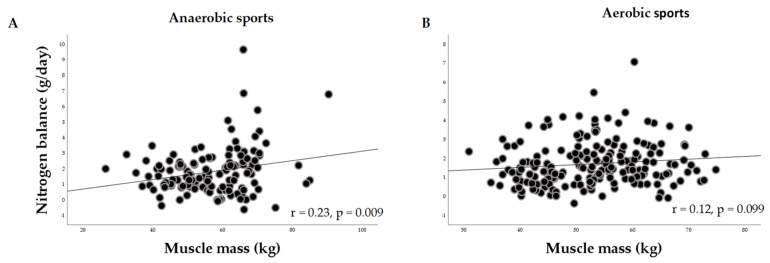
(**A**) The relationship between the NB (g/day) and the size of muscle mass (kg) in anaerobic male athletes (r = 0.23, *p* = 0.009); (**B**) the relationship between the NB (g/day) and the size of muscle mass (kg) in aerobic male athletes (r = 0.12, *p* = 0.1).

Figure 2 shows the relationship between the nitrogen balance (g/day) and the size of muscle mass (kg) in elite male athletes. Taking into consideration that the current study indicated a net 24-h positive nitrogen balance of 2 g in both anaerobic and aerobic male athletes, it can be summarized that a positive nitrogen balance during the periods of adequate nutrition was correlated with the potential promotion of muscle growth only in the cases where anaerobic training acted as a stimulus for nitrogen/protein retention (r = 0.23, *p* = 0.009).

**Figure 3:**
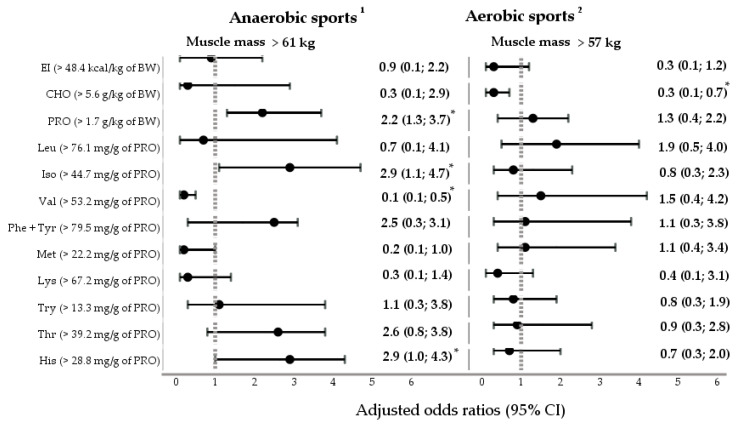
The association between the intake of macronutrients and essential amino acids (EAAs) and muscle mass (in kg) as a dependent variable in the samples of anaerobic and aerobic male athletes (multivariate analyses). OR_adj_—adjusted odds ratio (OR_adj_ = e^β^); 95% CI—95% confidence interval; *—*p*-value < 0.05. ^1^—reference category: muscle mass ≤ 61 kg; ^1^—Nagelkerke R^2^ = 0.59; the logistic regression model was adjusted for the age and training experience of anaerobic male athletes. ^2^—reference category: muscle mass ≤ 57; ^2^—Nagelkerke R^2^ = 0.29; the logistic regression model was adjusted for the age and training experience of aerobic male athletes. BW—body weight; EI—energy intake; CHO—carbohydrates; PRO—protein; Leu—leucine; Iso—isoleucine; Val—valine; Phe + Tyr—phenylalanine plus tyrosine; Met—methionine; Lys—lysine; Try—tryptophan; Thr—threonine; His—histidine.

The results of the multivariate analysis are displayed in Figure 3. Two multivariate logistic regression models were constructed to understand how the determinants related to dietary intake may predict muscle mass in the samples of anaerobic and aerobic elite male athletes. Multivariate logistic regression models were adjusted for the age and training experience of male athletes.

The ORs_adj_ for increased muscle mass were associated with higher intakes of protein (OR_adj_ 2.2; 95% CI: 1.3; 3.7) as well as EAAs such as isoleucine (OR_adj_ 2.9; 95% CI: 1.1; 4.7) and histidine (OR_adj_ 2.9; 95% CI: 1.0; 4.3) in a group of anaerobic athletes. In addition, an inverse association (OR_adj_ 0.1; 95% CI: 0.1; 0.5) was observed between higher intakes of valine and muscle development in anaerobic athletes.

In contrast, there was no relationship between the increased intake of protein or EAAs and the size of muscle mass in a sample of aerobic athletes. The results of this study revealed only the association (OR_adj_ 0.3; 95% CI: 0.1; 0.7) between the lower-carbohydrate diet and the decrease in muscle mass among well-trained and elite endurance male athletes.

## 4. Discussion

### 4.1. Athletic Body Composition

When body composition was one of the factors triggering exercise-induced adaptations, our study findings revealed both the high development of the musculoskeletal mass and the significant increase in the body fat mass percentage in both groups of Lithuanian male elite athletes. We compared our study’s empirical data with those described by other reports on the body composition assessment of professional male athletes through bioimpedanciometry published in Canada [59], the United States of America (USA) [60,61], Portugal [62], Australia [63], Spain [64,65], Turkey [66], Russia [67,68], Taiwan [69], Indonesia [70], Republic of Korea [71], Poland [72], and Czech [73]. The cohorts of professional male athletes from the target countries provided the mean outcomes for LBM (%), BF (%), and MFMI as follows: 84.2%, 14.9%, and 5.8 for male athletes engaged in anaerobic sports such as volleyball [59], football [60], judo [62,66], boxing [63], track and field [64], ski jumping [67], wrestling [69], and weightlifting [70], and 84.3%, 14.9%, and 5.2 for male athletes from aerobic sports, namely rowing [65], road cycling [61], cross-country skiing [71], biathlon [69], long-distance running [72], and pentathlon [73]. It should be highlighted that the mean MFMIs of the elite anaerobic male athletes we studied were lower than the MFMI values of well-trained sportsmen from foreign countries (5.2 (95%CI: 4.8; 5.8) vs. 5.8 (95% CI: 3.9; 7.6)). Thus, our study finding regarding the poorer body composition status can be explained only by the higher fat mass percentage (~17%) found in Lithuanian male athletes compared to the figures for the average fat levels of high-performance anaerobic athletes from other countries (~15%).

### 4.2. Nutritional Status of Athletes

The essential nutrients in the diets of studied Lithuanian elite male athletes were unbalanced due to an increased fat intake. A carbohydrate-deficient diet was found only among the athletes training for endurance-based sports. The study findings on low carbohydrate intakes of male athletes were consistent with the results obtained by other scientists who also revealed low carbohydrate consumption, which was felt to be significantly short of suggested levels for aerobic athletes [74,75,76,77].

An insufficient intake of carbohydrates by athletes can lead to the deterioration of aerobic capacity, may cause rapid fatigue during exercise, can lead to exercise-related immunodepression, and increase the risk of overtraining. As a result, muscle adaptations to aerobic training may be slowed down [78], coupled with performance impairments [79,80,81,82,83]. Therefore, this lack of a nutritional profile related to carbohydrate deficit among endurance male athletes should be corrected appropriately by converting a high-fat diet to a high-carbohydrate diet.

Also, it should be mentioned that both aerobic and anaerobic athletes adopted a diet high in protein. According to our study, the average amount of protein consumed by elite male athletes amounted to 1.8 g/kg/day and fully complied with the current international Recommended Dietary Allowance (RDA) for protein. Both the International Olympic Committee (IOC) and the International Society of Sports Nutrition (ISSN) have reported that, in order to maintain a positive nitrogen balance, professional athletes may benefit from approximately twice the RDA of protein (1.4–2.0 g/kg/day) [47,48]. A similar situation was observed among foreign professional athletes who had adopted a diet high in protein and adequately met recommendations for protein intake [1,31,32,59,84,85,86,87,88,89,90,91,92].

In addition, it is recommended that elite athletes should consume all EAAs with food in order to ensure the whole-body protein synthesis and to achieve maximum muscle mass gain. Our study showed that there were no differences in amino acid consumption in the samples of aerobic and anaerobic athletes. It should be highlighted that the uptake of amino acids for protein remodeling after the absorption process may vary due to the impact of the type of sport. Amino acids from extracellular space are used for muscle protein synthesis following resistance exercises. In contrast, new mitochondrial protein synthesis is maintained by the utilization of amino acids from intracellular space. Thus, there is no difference between the recommendations for resistance and endurance athletes in terms of the intake of protein/amino acids.

Furthermore, our analysis of the qualitative composition of amino acids consumed by anaerobic male athletes showed that the dietary intakes of all EAAs were significantly higher than the recommended ones. Moreover, this study focused on the intake of amino acid, namely leucine, as an essential stimulant of muscle protein synthesis. Although eating a diet of high-quality essential amino acid-rich protein containing 0.7 to 3.0 g (2–12 g/day) of leucine in evenly divided doses over the day (a schedule of every 3–4 h) is recommended by the IOC and ISSN [47,48,50]; hence, our study documented relatively high levels (10 g/day) of leucine intake in both anaerobic and aerobic sportsmen. The diets higher in leucine quantities (~10–14 g/day), although without significant correlation with the magnitude of muscle mass, were identified in our previous study dealing with the nutritional assessment of male athletes engaged in sports such as rowing, including canoeing and kayaking, too [93].

### 4.3. Nutritional Intake and Muscle Mass

#### 4.3.1. Carbohydrates for Muscle Development

This study found an association between the decreased size of muscle mass and the reduced intake of carbohydrates in a sample of aerobic athletes. The findings of the present study, related to the risk of a low-carbohydrate diet leading to a potentially negative effect on the synthesis of new myofibrillar proteins, can be explained by the fact that the ingestion of carbohydrates and amino acids immediately after exercise promotes the glucose-stimulated hormone insulin secretion and may theoretically inhibit the rate of protein degradation. However, a combined intake of carbohydrates and amino acids after exercise appears not to be any more a trigger for myofibrillar protein synthesis than the consumption of an amino acid mixture in an isolated form. Therefore, with the purpose of achieving training-induced muscle growth, only food high in protein/amino acids (with or without carbohydrates) for consumption is sufficient after exercise [94,95]. On the other hand, when a diet lacks carbohydrates, aerobic athletes exercise with decreased glycogen stores in their muscles and liver, a condition that can alter amino acid oxidative losses during exercise [96]. Thus, additional questions on protein requirements for endurance athletes that take into account the impact of carbohydrate availability still need to be addressed.

#### 4.3.2. Protein/EAAs for Muscle Development

This study demonstrated the association between a higher intake of protein and possible anaerobic training-induced muscle gain.

The scientific literature has well documented that the branched-chain amino acids (BCAAs) such as leucine, valine, and isoleucine correspond to almost 50% of muscle protein EAAs [5,6]. More specifically, leucine alone is capable of initiating the process of protein synthesis by triggering activation of the skeletal muscle mammalian target of rapamycin complex 1 (mTORC1), the protein complex that controls protein synthesis. After initiating this nutrient-sensing pathway, all amino acids are used in the synthesis of new muscle proteins [97]. However, the results of leucine intake (~10 g/day) in the male athletes we studied did not correlate with the scientific data on the outcomes of BCAA/leucine on muscle protein synthesis and possible muscle mass gain.

There are some explanations for these inconsistent findings. Firstly, as Santos et al. [98] speculated on the necessity of consumption of all EAAs to achieve optimal post-exercise muscle protein remodeling, supplementing a diet with isolated forms of BCAAs was no longer recommended for athletes [98]. In the second place, when consumed together, after absorption into the bloodstream, the BCAAs and other amino acids compete with each other for transport into the intestinal cells and myocytes. Therefore, single amino acid overconsumption may lead to the limitation of protein synthesis activation as a result of the decreased entrance of other EAAs into the muscle cells [99,100]. This type of pathway was assigned to explain the association between the increased muscle mass and higher intakes of isoleucine and histidine as well as to interpret our opposite study data concerning an inverse association between a higher valine intake and the decreased level of muscle hypertrophy in anaerobic male athletes. These findings can be explained by the likelihood that BCAAs such as valine and leucine were unbalanced in athletes’ diets due to higher intakes of isoleucine and histidine. More specifically, some documented evidence indicates that all of the BCAAs and other amino acids (histidine, tryptophan, tyrosine) are actively transferred into the human body cells via a neutral and identical heterodimeric membrane transport protein (LAT1) [101]. Thus, in this case, the consumption levels of both leucine and valine may not be balanced for benefiting the myofibrillar hypertrophy in the male athletes we studied.

However, histidine, unlike valine and leucine, has been shown to have a potential role in muscle hypertrophy in the anaerobic athletes we studied. This finding can be attributed to the fact that histidine is a precursor of dipeptide carnosine, which is composed of the two amino acids beta-alanine and histidine. Carnosine is an intracellular buffer that protects skeletal muscle cells from the damaging effects of acids (lactate and other acidic by-products of anaerobic metabolism). Consequently, the supplementation of the diets of athletes with histidine and/or beta-alanine may be an effective ergogenic aid for exercise lasting 1–4 min [101]. Attention must also be devoted to the evidence that due to a decrease in intracellular pH during exercise, the amino acid transmembrane transporter activity is also reduced, which can lead to reduced amino acid uptake into muscle cells while reducing the muscle protein synthesis rate following exercise [99].

### 4.4. Limitations

This comparative cross-sectional study coincided with several limitations. First, a limitation of this study was associated with the fact that the findings referring to the relationship between the nutritional profile and muscle mass quantities among professional athletes did not allow for causal conclusions. Therefore, cohort studies in design together with randomized controlled trials (RTCs) are warranted to identify the effect of the optimal dietary amino acid ratio on body composition, especially in athletic populations.

Second, our study findings are only applicable to elite male athletes and might not be generalizable to other populations. Unfortunately, there is a lack of research on how to optimize nutrition for females in terms of the disparities in health and physiology. In terms of female-specific physiology for athletes, the findings of male athletes may be misapplied to female athletes [102,103]. It was “more difficult” to recruit females for the study due to higher hormonal intricacy [104], possible risk of low energy availability [105], and the female athlete triad [106]; therefore, sportswomen were not included in our research. Future research must focus on a cohort of high-power and high-endurance female athletes with distinct body composition characteristics and metabolic profiles [107].

Third, it has been widely documented that self-reported dietary intakes can result in biases due to underestimated outcomes, as observed in a number of cohorts, including adolescents [108], overweight persons [109], and athletes [110,111,112]. Also, despite the food records approach being considered a "gold standard" in nutritional status assessment, it creates difficulties for participants to document information correctly and accurately, as well as to trust that researchers will correctly encode the data using the relevant databases [113,114,115]. Along these lines, three non-consecutive day diet logs derived from single administrations of 24-hour dietary records were incapable of accounting for day-to-day variation and potentially contributed to trivial biases of empirical data interpretation.

Fourth, although the DXA preferred as a "gold standard" approach leads only to an average accuracy in estimating body fat levels, and there is no gold-standard body composition analysis technique [84,85,86,87,88,89,90,91,92,93,94,95,96,97,98,99,100,101,102,103,104,105,106,107,108,109,110,111,112,113,114,115,116,117,118,119,120,121,122], the bioelectrical impedance analysis (BIA) method referring to a third level of validity has been constructed [22]. Furthermore, scientific evidence refers to very strong correlations between the DXA and different BIA models [23]. Thus, in our case, the body composition assessments were limited to BIA. Conclusively, the application of the DXA method with higher levels of consistency and lower biases is needed to match our outcomes with the male athlete body composition results reported by other scientific researchers [123].

Finally, although our study focused on the adjusted odds ratios of dietary macronutrients/EAAs for musculoskeletal mass, muscle development is multivariable and may be determined by more potential factors such as the specificity of sports disciplines, training phases and periods within a macrocycle, intensities used during training loads, exercise-induced adaptation levels, etc.

## 5. Conclusions

All elite male athletes showed the consumption of lipids above the recommended levels. The present study highlighted a significant macronutrient imbalance due to decreased carbohydrate intake in a group of aerobic male athletes. Notably, research by Syed-Abdul et al. [124] explored the effects of self-implemented carbohydrate cycling and resistance exercise on body fat reduction in anaerobic athletes, shedding light on the connection between dietary strategies and body composition changes.

The mean daily protein intake of 1.8 g/kg/day was optimal for anabolism in the samples of both anaerobic and aerobic male athletes. Nevertheless, protein intake in appropriate doses was associated with a potential muscle mass gain only in anaerobic male athletes. Regardless of sufficient levels of anabolism-inducing protein consumption, our study revealed the relationship between a carbohydrate-deficient diet and the decrease in muscle mass among aerobic male athletes.

Since sufficient availability of all amino acid precursors is a prerequisite for increasing the rate of protein synthesis in muscles, the findings of this study disclosed the positive association between the possible muscle mass gain and the increased intakes of amino acids such as isoleucine and histidine among anaerobic athletes. Our study also revealed an inverse relationship between a higher intake of valine and a decreased level of feasible muscle hypertrophy in anaerobic athletes. Evidently, in terms of the quality of dietary amino acid composition, the increased intake of essential amino acids such as isoleucine and histidine may potentially contribute to imbalances in diet quality, and this type of imbalance may determine the intramuscular availability of other essential amino acids following the absorption process.

Finally, this study highlighted that dietary amino acid composition is as important as the total protein intake. Therefore, it is recommended that sports dieticians organize meal plans for athletes that are not only adjusted to the total protein content, but optimized for dietary amino acid composition, too. More importantly, taking into consideration the generalized data of this study, the recommendations for sports nutritionists should emphasize the necessity of nutrition assessment and recommendations for sportsmen regarding their dietary strategies on how to manipulate dietary amino acids composition with respect to achieving long-term body composition goals. However, alterations in muscle cells may not lead to the whole-body response to physical loads. Consequently, the benefits of dietary amino acid balance for muscle development along with physical adaptation should be considered only as probabilistic.

## Figures and Tables

**Table 1:** Distribution of male athletes (in percentage) engaged in different sports according to the dominant energy-producing pathway in the body.

Anaerobic Sports	Eligible		Analyzed		Aerobic Sports	Eligible		Analyzed	
	n = 138		n = 104			n = 198		n = 130	
	n	%	n	%		n	%	n	%
Boxing	15	10.9	14	13.5	Rowing	37	18.7	28	21.5
Judo	13	9.4	6	5.8	Road cycling	51	25.8	31	23.8
Greco-Roman wrestling	30	21.7	29	27.9	Swimming	44	22.2	29	22.3
Taekwondo	4	2.9	3	2.9	Skiing	19	9.6	12	9.2
Weightlifting	8	5.8	6	5.8	Biathlon	22	11.1	17	13.1
Basketball	53	38.4	39	37.5	Long-distance running	13	6.6	8	6.2
Gymnastics	4	2.9	2	1.9	Modern pentathlon	12	6.1	5	3.8
Disc throw, javelin throw	7	5.1	3	2.9	-			-	-
High jump	4	2.9	2	1.9	-			-	-

**Table 2:** Training programs for elite male athletes.

Variables	Anaerobic Sports (n = 104)	Aerobic Sports (n = 130)
Training period	Special training	Special training
Training experience, years	8.2 ± 3.7	7.8 ± 3.9
Exercise per month, days	23.3 ± 3.1	23.1 ± 3.6
Duration of training, hours per month	41.9 ± 12.1	48.6 ± 17.4
Duration of training, hours per day	2.8 ± 1.1	3.1 ± 1.1
Physical activity levels were allocated for five intensity zones depending on energy-producing pathway during workouts (% ^1^)		
Aerobic endurance training, recovery: heart rate equaled 130 ± 10 bpm, blood lactate levels were up to 2 mmol/L	10–17%	17–44%
Aerobic strength training: heart rate equaled 150 ± 10 bpm, blood lactate levels were 2–4 mmol/L, and special muscular power increased at the anaerobic threshold	19–41%	37–70%
Aerobic and anaerobic glycolytic strength training: heart rate equaled 170 ± 10 bpm, blood lactate levels were 4–12 mmol/L	13–36%	9–34%
Anaerobic glycolytic strength training: heart rate ≥ 181 bpm, blood lactate levels were up to 21 mmol/L	0–6%	0–7%
Anaerobic phosphocreatine strength training: blood lactate levels were 1.5–6 mmol/L	0–2%	0–4%

bpm—beats per minute; ^1^—time allocated for intensity areas during workouts (%).

**Table 3:** Body composition of male athletes depending on sports.

Variables	Anaerobic Sports (n = 104)	Aerobic Sports (n = 130)	Normative Data	∆_ana_ (95% CI)	*D*	∆_aer_ (95% CI)	*d*
Height (m)	1.8 ± 0.2	1.8 ± 0.1	-	-	-	-	-
Body weight (kg)	77.5 ± 17.4	75.1 ± 11.6	-	-	-	-	-
Lean body mass (kg)	63.8 ± 11.4	62.2 ± 7.7	-	-	-	-	-
Lean body mass (% of BW)_BIA_	83.2 ± 5.3	83.3 ± 4.2	75–85 ^b^ (80 ^a^)	3.3 (2.2; 4.3)	0.6	3.3 (2.6; 4.1)	0.8
Muscle mass (kg)	59.3 ± 10.5	57.9 ± 7.0	-	-	-	-	-
Muscle mass (% of BW)_BIA_	77.4 ± 5.2	77.6 ± 4.1	74–80 ^c^ (77 ^a^)	0.4 (−0.6; 1.4)	0.1	0.6 (−0.1; 1.3)	0.1
Body fat (kg)	13.7 ± 7.1	12.9 ± 4.7	-	-	-	-	-
Body fat (% of BW)_BIA_	16.7 ± 5.2	16.7 ± 4.2	10–14 ^d^ (12 ^a^)	4.7 (3.7; 5.7)	0.2	4.7 (3.9; 5.4)	0.2
Muscle and fat mass index	5.3 ± 2.4	5.2 ± 2.6	4.7–6.0 ^e^ (5.4 ^a^)	–0.1 (−0.5; 0.4)	0.02	−0.2 (−0.6; 0.3)	0.05

Data are presented as means ± SD; SD—standard deviation; BW—body weight; ∆—delta; ∆_ana_—actual measure of anaerobic athletes–recommended levels for anthropometric measurements; ∆_aer_—actual measure of aerobic athletes–recommended levels for anthropometric measurements; 95% CI—95% confidence intervals; *d*—effect size (*d*); ^a^—measure of central tendency; ^b^—lean body mass norm for male is 75–85%; ^c^—muscle mass norm for male is 74–80%; ^d^—optimal body fat for male athletes is 10–14% [26]; ^e^—extensive muscle and fat mass index for male athletes is 4.7–6.0 [26].

**Table 4:** Nutritional profile and nitrogen balance of male athletes according to sports.

Nutrient Intake and Nitrogen Balance	Anaerobic Sports (n = 104)	Aerobic Sports (n = 130)	Requirements	∆_ana_ (95% CI)	*d*	∆_aer_ (95% CI)	*d*
Energy intake, kcal/kg/day	50 ± 15.9	49.6 ± 12.8	55.6 ± 4.2	−6.3 (−8; −4.6)	0.5	−6.2 (−8.2; −4.2)	0.6
Fat, %	39.6 ± 6.9	40.3 ± 8.2	25–35 (30 ^a^)	10.3 (8.8; 11.7)	1.4	9.6 (8.3; 10.9)	1.3
Carbohydrates, g/kg/day	5.6 ± 1.9	5.7 ± 0.6	7–10 (8.5 ^a^)	−2.9 (−3.5; −3.3)	1.5	−2.9 (−3.2; −2.5)	4.7
Protein, g/kg/day	1.8 ± 0.7	1.8 ± 0.6	1.4–2.0 (1.7 ^a^)	0.1 (–0.1; 0.2)	0.1	0.1 (−0.1; 0.2)	0.1
Leu, mg/g of protein/day	76 ± 6.6 ^1^	76 ± 5.8 ^2^	59 ^3^	16.8 (15.5; 18)	2.6	17 (15.9; 18)	2.9
Iso, mg/g of protein/day	45 ± 3.2 ^1^	45 ± 3.4 ^2^	30 ^3^	14.5 (13.8; 15)	4.7	14.7 (14; 15.3)	4.4
Val, mg/g of protein/day	53 ± 4.3 ^1^	53 ± 3.8 ^2^	39 ^3^	14.1 (13.2; 15)	3.3	13.7 (13; 14.4)	3.3
Phe + Tyr, mg/g of protein/day	80 ± 6.8 ^1^	79 ± 6.5 ^2^	38 ^3^	41.7 (40.4; 43)	6.2	41.5 (40; 42.6)	6.3
Met, mg/g of protein/day	22 ± 2.2 ^1^	22 ± 2.2 ^2^	16 ^3^	5.9 (5.5; 6.3)	2.7	6.2 (5.8; 6.5)	2.7
Lys, mg/g of protein/day	67 ± 5.9 ^1^	67 ± 6.2 ^2^	45 ^3^	21.8 (21; 22.9)	3.7	21.7 (20.7; 23)	3.5
Try, mg/g of protein/day	13 ± 1.5 ^1^	13 ± 1.4 ^2^	6 ^3^	7.1 (6.8; 7.4)	4.7	7.4 (7.2; 7.7)	5
Thr, mg/g of protein/day	39 ± 2.8 ^1^	39 ± 2.7 ^2^	23 ^3^	16 (15; 16.6)	5.7	16.4 (16; 16.9)	5.9
His, mg/g of protein/day	28 ± 3.2 ^1^	28 ± 2.9 ^2^	15 ^3^	13.2 (12.7; 14)	4.1	13.4 (13; 13.9)	4.5
24 h nitrogen balance, g	2.0 ± 1.6	1.9 ± 1.1	≥1	0.9 (0.7; 1.1)	0.6	9.6 (8.3; 10.9)	0.8

Data are presented as means ± SD; SD—standard deviation; ∆—delta; ∆_ana_—actual nutrient intake and nitrogen balance in anaerobic athletes—recommended levels for nutrient intake and nitrogen balance; ∆_aer_—actual nutrient intake and nitrogen balance in aerobic athletes–recommended levels for nutrient intake and nitrogen balance; 95% CI—95% confidence intervals; *d*—effect size (*d*); ^a^—measure of central tendency; ^1^—essential amino acid (EAA) (mg/g of protein) intake in anaerobic athletes; ^2^—EAA (mg/g of protein) intake in aerobic athletes; ^3^—EAA requirements (mg/g of protein). EAA requirements: the best current estimates have been reported by the World Health Organization (WHO) [49]. EAA requirements for athletes have been assessed in accordance with the recommendations made by the WHO, following an empirical recalculation in accordance with the standards applicable to athletes [47,48,50]; Leu—leucine; Iso—isoleucine; Val—valine; Phe + Tyr—phenylalanine plus tyrosine; Met—methionine; Lys—lysine; Try—tryptophan; Thr—threonine; His—histidine.

## Data Availability

Data are available on request.
